# Chemistry and crystal engineering

**DOI:** 10.1107/S2052252524006249

**Published:** 2024-06-28

**Authors:** Susan A. Bourne

**Affiliations:** a Centre for Supramolecular Chemistry Research, Department of Chemistry University of Cape Town Rondebosch 7701 South Africa

**Keywords:** crystal engineering, non-covalent interactions, editorial

## Abstract

Recent studies published in the *Chemistry and crystal engineering* section of **IUCrJ** emphasize developments both in methodology and techniques as well as the diverse range of classes of compounds being studied and of problems being tackled.

The **IUCrJ** theme of *Chemistry and crystal engineering* covers a broad range of topics and this is reflected in the articles published in **IUCrJ**. In 2022, Desiraju suggested that ‘the Holy Grail of crystal engineering is to answer the question: *Given the molecular structure of a compound what are all the possible crystal structures?*’ (Desiraju, 2022 ▸). It is interesting and instructive therefore to reflect on advances made towards this goal in recent years. While there is still a focus in crystal engineering to deepen our understanding of the influence of intermolecular interactions on crystal packing, increasingly the focus has moved to practical applications such as devices and materials with useful physical and chemical properties (Braga, 2023 ▸; Xiong *et al.*, 2024 ▸; Kasuya *et al.*, 2024 ▸; Xu *et al.*, 2024 ▸). Recent articles in the journal highlight developments both in methodology and techniques as well as a diverse range of classes of compounds being studied and of problems being tackled.

Studies on the nature of non-covalent interactions are highly topical (Biot & Bonifazi, 2020 ▸; Rajapaksha *et al.*, 2023 ▸) and are well represented in recent publications in the journal. These include the nature of π-hole interactions between iodide anions and quinoid rings which have been shown to be predominantly electrostatic (Milašinović *et al.*, 2023 ▸) while Carroll and co-workers used a crystalline sponge to isolate and characterize a family of bi­aryl alcohol guest molecules, showing that the orientation and molecular conformation adopted by the guest molecules is controlled by the intermolecular interactions (hydrogen-bonding, halogen-bonding and/or aromatic interactions) available at the guest exchange site (Carroll *et al.*, 2023 ▸).

Interesting questions remain, such as how far can a packing motif change while still being considered the same? This is the subject of a re-evaluation of the definition of isostructurality (Bombicz, 2024 ▸) which argues that consideration of symmetry and supramolecular interactions and their influence on the resulting crystal structure is required. The concept of similarity and isostructurality is key to the study of polymorphism (Subanbekova *et al.*, 2024 ▸; Jeziorna *et al.*, 2023 ▸) and even of the disappearance and reappearance of polymorphs (Sacchi *et al.*, 2024 ▸). A recent example of the value of such considerations is the study of the mechanism and kinetics of transforming polymorph C of the pharmaceutical ingredient ibrutinib to its halogen benzene solvates using a flow-through capillary mounted directly on a powder X-ray diffractometer (Jirát *et al.*, 2023 ▸). Four different transformation mechanisms were determined despite the isostructurality of three of the four solvated crystal structures. The influence of environmental factors on molecular packing is another area of ongoing interest. The increasing availability of diamond-anvil cells has promoted an interest in the effect of pressure on molecular packing. High pressure promotes some interactions over others and can force the molecules to adopt a particular conformation (Sacharczuk *et al.*, 2024 ▸) while pressure changes can induce migration of a proton to allow interconversion between a cocrystal form to an ionic salt (Patyk-Kaźmierczak *et al.*, 2024 ▸).

Crystallography may be said to have reached a mature phase as a science but new techniques and computational approaches are essential to allow researchers to tackle ever more complex problems. Recent developments in quantum crystallography hold great promise for improving the accuracy of key structural parameters. For example, Hirshfeld atom refinement (HAR) uses aspherical structure factors obtained from calculation to produce more accurate structural information such as more accurate hydrogen atom positions even in cases where the hydrogens are close to heavy elements (Capelli *et al.*, 2014 ▸; Woińska *et al.*, 2023 ▸). Zwolenik *et al.* (2024 ▸) applied HAR to a molecular structure at high pressure and compared the result with an analysis of the same structure measured over a temperature range of 90 to 390 K.

Developments in machine learning and artificial intelligence will have an increasing impact on the nature of the questions that can be addressed using crystallography (Ekeberg, 2024 ▸; Billinge & Proffen, 2024 ▸; Zhu *et al.*, 2024 ▸). An intriguing example is recent work by Takiguchi *et al.* who devised a deep learning model to determine which of 20000 metal complexes extracted from the Cambridge Structural Database (CSD) are single-molecule magnets (SMM) with 70% accuracy (Takiguchi *et al.*, 2024 ▸). The latter article highlights the enormous value the CSD (Ferrence *et al.*, 2023 ▸) and other databases offer so it is encouraging to see the work being done by CODATA to ensure the interoperability of crystallographic data within the FAIR principles (findable, accessible, interoperable and reusable) (Brink *et al.*, 2024 ▸).

In conclusion, recent articles in the journal are evidence that crystal engineering concepts are well embedded in many aspects of chemical crystallography. Further advances in these themes are to be expected and will be welcomed in **IUCrJ**.

## References

[bb1] Billinge, S. J. L. & Proffen, Th. (2024). *Acta Cryst.* A**80**, 139–145. 10.1107/S2053273324000172 38275223

[bb2] Biot, N. & Bonifazi, D. (2020). *Chem. Eur. J.* **26**, 2904–2913. 10.1002/chem.201904762 31840314

[bb3] Bombicz, P. (2024). *IUCrJ*, **11**, 3–6. 10.1107/S2052252523010436 PMC10833389 38096040

[bb4] Braga, D. (2023). *Chem. Commun.* **59**, 14052–14062. 10.1039/d3cc04313d 37938038

[bb5] Brink, A., Bruno, I., Helliwell, J. R. & McMahon, B. (2024). *IUCrJ*, **11**, 9–15. 10.1107/S2052252523010424 PMC10833386 38131388

[bb6] Capelli, S. C., Bürgi, H.-B., Dittrich, B., Grabowsky, S. & Jayatilaka, D. (2014). *IUCrJ*, **1**, 361–379. 10.1107/S2052252514014845 PMC4174878 25295177

[bb7] Carroll, R. C., Harrowven, D. C., Pearce, J. E. & Coles, S. J. (2023). *IUCrJ*, **10**, 497–508. 10.1107/S2052252523005146 PMC10324488 37409807

[bb8] Desiraju, G. R. (2022). *IUCrJ*, **9**, 329–330. 10.1107/S2052252522004274 PMC9067120 35546803

[bb9] Ekeberg, T. (2024). *J. Appl. Cryst.* **57**, 1–2. 10.1107/S1600576723010476 PMC10840311 38322721

[bb10] Ferrence, G. M., Tovee, C. A., Holgate, S. J. W., Johnson, N. T., Lightfoot, M. P., Nowakowska-Orzechowska, K. L. & Ward, S. C. (2023). *IUCrJ*, **10**, 6–15. 10.1107/S2052252522010545 PMC9812213 36598498

[bb11] Jeziorna, A., Paluch, P., Zając, J., Dolot, R. & Dudek, M. K. (2023). *Cryst. Growth Des.* **23**, 5998–6010.

[bb12] Jirát, J., Rohlíček, J., Kaminský, J., Jirkal, T., Ridvan, L., Skořepová, E., Zvoníček, V., Dušek, M. & Šoóš, M. (2023). *IUCrJ*, **10**, 210–219. 10.1107/S2052252523001197 PMC9980385 36815712

[bb13] Kasuya, K., Oketani, R., Matsuda, S., Sato, H., Ishiwari, F., Saeki, A. & Hisaki, I. (2024). *Angew. Chem. Int. Ed.* **63**, e202404700. 10.1002/anie.202404700 38577718

[bb14] Milašinović, V., Vuković, V., Krawczuk, A., Molčanov, K., Hennig, C. & Bodensteiner, M. (2023). *IUCrJ*, **10**, 156–163. 10.1107/S2052252523000052 PMC9980391 36692857

[bb15] Patyk-Kaźmierczak, E., Izquierdo-Ruiz, F., Lobato, A., Kaźmierczak, M., Moszczyńska, I., Olejniczak, A. & Recio, J. M. (2024). *IUCrJ*, **11**, 168–181. 10.1107/S2052252524000344 PMC10916288 38275161

[bb16] Rajapaksha, H., Augustine, L. J., Mason, S. E. & Forbes, T. Z. (2023). *Angew. Chem. Int. Ed.* **62**, e202305073. 10.1002/anie.202305073 37177866

[bb17] Sacchi, P., Wright, S. E., Neoptolemou, P., Lampronti, G. I., Rajagopalan, A. K., Kras, W., Evans, C. L., Hodgkinson, P. & Cruz-Cabeza, A. J. (2024). *Proc. Natl Acad. Sci. USA*, **121**, e2319127121. 10.1073/pnas.2319127121 PMC11009673 38557191

[bb18] Sacharczuk, N., Olejniczak, A., Bujak, M., Dziubek, K. F., Katrusiak, A. & Podsiadło, M. (2024). *IUCrJ*, **11**, 57–61. 10.1107/S2052252523009995 PMC10833391 38019132

[bb19] Subanbekova, A., Bezrukov, A. A., Bon, V., Nikolayenko, V. I., Koupepidou, K., Sensharma, D., Jan Nikkhah, S., Wang, S.-Q., Kaskel, S. & Vandichel, M. (2024). *ACS Appl. Mater. Interfaces*. In the press. 10.1021/acsami.4c03777 PMC11082895 38666365

[bb20] Takiguchi, Y., Nakane, D. & Akitsu, T. (2024). *IUCrJ*, **11**, 182–189. 10.1107/S2052252524000770 PMC10916298 38299376

[bb21] Woińska, M., Pawlędzio, S., Chodkiewicz, M. L. & Woźniak, K. (2023). *J. Phys. Chem. A*, **127**, 3020–3035. 10.1021/acs.jpca.2c06998 PMC10084459 36947670

[bb22] Xiong, Y.-A., Duan, S.-S., Hu, H.-H., Yao, J., Pan, Q., Sha, T.-T., Wei, X., Ji, H.-R., Wu, J. & You, Y. (2024). *Nat. Commun.* **15**, 4470. 10.1038/s41467-024-48948-0 PMC11127950 38796520

[bb23] Xu, W., Huang, G., Yang, Z., Deng, Z., Zhou, C., Li, J.-A., Li, M.-D., Hu, T., Tang, B. Z. & Phillips, D. L. (2024). *Nat. Commun.* **15**, 2561. 10.1038/s41467-024-46869-6 PMC10959985 38519517

[bb24] Zhu, Z., Zhang, Y., Wang, Z., Tang, W., Wang, J. & Gong, J. (2024). *Cryst. Growth Des.* **24**, 4245–4270.

[bb25] Zwolenik, A., Tchoń, D. & Makal, A. (2024). *IUCrJ*, **11**, 519–527. 10.1107/S2052252524003634 PMC11220879 38727170

